# Sexual Satisfaction and Sexual Reactivity in Infertile Women: The Contribution of The Dyadic Functioning and Clinical Variables 

**DOI:** 10.22074/ijfs.2015.4604

**Published:** 2015-12-23

**Authors:** Anna Czyżkowska, Katarzyna Awruk, Konrad Janowski

**Affiliations:** Faculty of Psychology, University of Finance and Management in Warsaw, Warsaw, Poland

**Keywords:** Infertility, Female, Sexuality, Sexual

## Abstract

**Background:**

Infertility is a factor which has been linked to higher prevalence of sexual dysfunctions in women; however, ambiguous results have been reported about the impact of infertility on women’s sexual satisfaction. The purpose of this study was to compare sexual and dyadic functioning in infertile and fertile women. Furthermore, the associations between sexual variables and clinical variables (depressive symptoms, period trying to conceive, and treatment period) were assessed in infertile women sample.

**Materials and Methods:**

The cross-sectional study involved 50 women with the history of infertility and 50 fertile women recruited from the general population. The Sexual Satisfaction Scale (SSS), Mell-Krat Scale (women’s version), Family Assessment Measure (FAM-III), and Beck Depression Inventory (BDI) were administered to all participants.

**Results:**

Infertile women reported lower sexual satisfaction and more maladaptive patterns of dyadic functioning in comparison to the control group. As many as 45 (90%) of infertile women, compared to 13 (26%) of the control group, reported the scores on the Mell-Krat Scale indicative of the presence of dysfunctions in sexual reactivity (P≤0.001). Infertile women reported significantly higher levels of depressive symptoms than the women from the control group (P≤0.001). Negative correlations were observed between sexual satisfaction and dyadic functioning in both groups (P≤0.05); however, the patterns of these associations were different in infertile and fertile women. For example, negative correlations were found between satisfaction with control and task accomplishment, role performance, affective involvement, and values and norms in infertile women. However, these relationships were not observed in the control group. No correlations were revealed between sexual reactivity and dyadic functioning in infertile women and the control group. Negative correlations were observed between satisfaction with control and relationship duration and treatment period as well as between sexual reactivity and period of trying to conceive. Multiple regression analyses also revealed different predictors of sexual satisfaction in both groups: affective involvement (P≤0.05) and relationship duration (P≤0.05) in infertile women, whereas communication (P≤0.05), affective expression (P≤0.05) and depressive symptoms (P≤0.05) in the control group.

**Conclusion:**

Infertility is an important factor affecting sexual and dyadic functioning and is linked to higher depressive symptoms in infertile women.

## Introduction

Infertility is a socio-medical problem affecting couples all over the world. Infertility is defined as 'the inability to conceive after 12 months of regular unprotected sexual intercourse' (1). The number of infertile women is constantly increasing. It has been estimated that approximately 72.4 million women worldwide are infertile (2). The prevalence rate of infertility in Polish women varies from 10 to 15% (3), whereas 13-17% of women in Finland are affected by infertility (4). Similarly, high prevalence rates of infertility have been reported in other countries such as Turkey (10%), USA (10%) and China (7-10%) (5). Taking into account the high prevalence of infertility and its psychological, social and economic consequences, it is important to realize that infertility is not only a common medical problem, but also an acute psychosocial issue (6).

Hence, numerous studies have been devoted to investigating causes of infertility (7-9) and its psychological implications (10, 11). For instance, it has been demonstrated that learning about one’s infertility is one of the most stressful experiences, comparable to those associated with the diagnosis of cancer, hypertension and human immunodeficiency virus (HIV)-positive status (12). Parenthood is believed to be not only a stage of life but also one of the most important developmental tasks with numerous implications for the relationships in the couple and within the whole family system. Therefore, unwanted infertility can be the cause of severe distress in infertile couples and the futile efforts to conceive exert long-term effects on human functioning.

Tao et al. (13) reported that infertility may have a negative influence on sexual behavior; however, this effect depends on gender, cause of infertility, duration of infertility and treatment success. For example, men revealed lower sexual satisfaction than women, especially when the male factor infertility was involved, whereas women had lower sexual satisfaction after an *in vitro* fertilization treatment that had been unsuccessful. Similarly, another study carried out among 200 infertile couples and 200 fertile couples indicated that infertile couples demonstrated lower self-esteem, marital satisfaction and sexual satisfaction (14) and it is supported by other findings indicating that infertility lasting for 3-6 years was linked to the highest relationship instability and the lowest sexual satisfaction (3). Lower sexual satisfaction may be caused by a decreased frequency of intercourse and it can be associated with sexual dysfunctions such as problems with sexual arousal (in infertile women), premature ejaculation and erection dysfunction (in infertile men) (13). The findings obtained in another study conducted among 308 infertile women and 308 fertile women indicated that 61.7% of infertile women had sexual dysfunctions compared to 26.55% of those without infertility. The dysfunctions involved desire, arousal and orgasm problems (5).

Moreover, it has been demonstrated that stress can play a detrimental role in the functioning and longevity of a close relationship (15). For instance, Bodenmann et al. (16) proposed the stress-divorcemodel, in which he suggested that chronic stress has an impact on marital satisfaction by decreasing the time that partners spend together, which in turn results in reduction of shared experiences, decreasing the quality of communication, increasing the likelihood that problematic personality traits will be expressed between partners (rigidity, anxiety, and hostility) and increasing the risk of psychological and physical problems, such as sleep disorders, sexual dysfunctions and mood disturbances.

Indeed, previous studies indicated that infertile couples have a higher level of depression than fertile couples (17). Likewise, childless women had an increased risk of dysthymia and anxiety disorders compared to women with children. Interestingly, women who currently have a child but experienced infertility in the past have a higher risk of developing a panic disorder (4). Among variables which may contribute to the development of depression and anxiety in infertile women, researchers found such factors as duration of infertility, cause of infertility, educational level (18), age, male factor infertility (19) and pressure from family (20). High rates of depressive symptoms among infertile women may also have an important impact on sexual activity and satisfaction. It has been demonstrated extensively that depressive symptoms are associated with impairments of sexual function and satisfaction (21) by inhibited sexual arousal, inhibited orgasm and less pleasure experienced during intercourse (22).

Taking into account the inconsistency of the findings from previous studies, it is essential to continue research on sexual functioning among infertile women.

Thus, in this study, we concentrated on comparison of infertile and fertile women with regard to sexual and dyadic functioning. Additionally, the associations between sexual satisfaction and dyadic functioning in infertile and control group as well as relationships between sexual satisfaction and clinical variables (depressive symptoms, period trying to conceive and treatment period) in infertile women sample were evaluated.

Thus, the present study aimed at i. Comparing sexual and dyadic functioning in infertile and fertile women samples and ii. Determining the relationship between sexual satisfaction/reactivity and relational and clinical variables in Polish infertile women.

## Materials and Methods

The study had a cross-sectional design and was conducted between November 2011 and February 2012. One-hundred women were recruited to participate in the study, including 50 infertile women and 50 women without known infertility diagnosis (control group). All the infertile women participated in the reproductive treatment in a private infertility outpatient clinic in Warsaw, Poland. The inclusion criteria for participation were: i. A confirmed diagnosis of infertility on the part of the women made by a gynecologist, ii. Ineffective efforts to conceive undertaken for a period of at least one year prior to the study, iii. Age between 18 and 40 years, iv. No children and v. Staying in an intimate relationship with a partner. Exclusion criteria included: i. The co-occurrence of serious chronic somatic diseases (such as diabetes, hyperthyroidism), ii. The co-occurrence of psychiatric disorders involving delusions or hallucinations and iii. Lack of consent to participate. The women fulfilling these criteria were approached by their treating doctor with the invitation to enroll into the study during their pre-scheduled visits in the clinic. A total of 79 women were initially approached, out of whom 50 agreed and gave their written informed consent to participate in the study. The control group consisted of 50 women recruited from the general population, who reported that they had never been treated for infertility and did not experience infertility-related problem. The inclusion/ exclusion criteria for the women from the control group were the same as for the patients, except for the known infertility diagnosis. To make the both groups comparable, the women from the control group were also required not to have children; however, they reported they did not have infertility-related problems. The women from the control group were recruited through announcements made by a local university newspaper and through the Internet. The women from the control group were offered an incentive in the form of a book. Initially, 92 women responded to the announcements, out of whom 50 fulfilled the required inclusion/exclusion criteria and showed up for a scheduled appointment to complete the questionnaires. After thorough explanation of the objective and nature of the study, written informed consent was obtained from all participants. The project of the study was approved by the University Ethical Committee, University of Finance and Management in Warsaw, ul. Pawia, Poland.

All participants completed a battery of questionnaires measuring sexual satisfaction, sexual reactivity, depressive symptoms and the qualities of dyadic functioning in their close relationship. All the instruments were standard psychological or sexological measures which had previously been validated in the Polish population. Additionally, socio-demographic and clinical data were collected by means of the Personal Data Sheet, designed for the purpose of this study. The infertile women filled in the questionnaires individually, in a separate room in the clinic, supervised by one of the researchers, a qualified psychologist. The women from the control group were invited to come to the University and completed the questionnaires individually in a separate room at the University during a pre-scheduled appointment, supervised by the same researcher.

The following instruments were used in the study:

Sexual Satisfaction Scale (SSS) is a self-report questionnaire developed by Davis et al. (23) to assess sexual satisfaction. The SSS consists of 21 items designed. All items are affirmative statements in the first person singular and they constitute three subscales: physical satisfaction, emotional satisfaction and satisfaction with control. The physical satisfaction subscale measures satisfaction with fulfillment of sexual needs, quality of the couple’s sexual contacts and the partners’ sexual abilities. The emotional satisfaction subscale is designed to evaluate satisfaction with the feelings towards the partner and his/her sexual behaviors. The satisfaction with control subscale serves to assess the control over one’s own sexual performance and frequency and timing of sexual intercourses. The responses are given on a 5-point Likert scale anchored with: I definitely disagree, I don’t agree, I am not sure/It is difficult to decide, I agree, I definitely agree. The scores are computed for each subscale. Higher scores indicate higher sexual satisfaction. High or satisfactory reliability coefficients (Cronbach’s α) were reported for the instrument: 0.85 for physical satisfaction, 0.84 for emotional satisfaction and 0.75 for satisfaction with control.

Mell-Krat Scale (the version for women) is a selfreport questionnaire measuring sexual reactivity (24, 25). Mell-Krat Scale consists of 20 multiple-choice items measuring a range of psychophysiological characteristics related to sexual reactivity like orgasm frequency, libido, intercourse orgasm frequency, pre-coitus arousal, vagina contractions during orgasm, etc. Each item describes five qualitatively different states related to a given aspect of sexual reactivity, ranging from highly disordered to fully satisfactory states. The items are scored on a 5-point scale, from 0 to 4. The total score is computed by summing up the scores for all items. The theoretical range of the scores is from 0 to 80, with lower scores interpreted as indicative of problems in sexual reactivity. The score of 55 was proposed as the cut-off for the recognition of sexual disorders. The reliability coefficient (Cronbach’s α) of 0.92 was reported for the Scale.

Family Assessment Measure (FAM-III) is a complex measure developed by Skinner et al. (26, 27). The Polish adaptation of the instrument was performed by Beauvale et al. (28). FAM-III consists of three questionnaires (the general scale, the self-rating scale and the dyadic relationship scale) used to evaluate family functioning. The general scale captures the family in terms of a system and it generally serves to examine general family functioning and its overall health. The self-rating scale is used to evaluate one’s own functioning within the family system. In the present study, only the scores from the third scalethe dyadic relationship-were used for further statistical analyses. This scale consists of 28 items designed to assess a particular relationship within the family system, for instance, an individual’s relationship with another family member. The items of this scale pertain to seven dimensions of family functioning: task accomplishment, role performance, communication, affective expression, affective involvement, control, and values and norms. The task accomplishment is the most important dimension in the process model as it allows to achieve biological, psychological and social goals by task fulfillment within the family system. The role performance dimension grasps the couple’s functioning, especially when the couple is faced with the need of tackling a common task. To facilitate task accomplishment and role performance, two other processes (communication and affective expression) are used. The affective involvement dimension refers to the degree and quality of the couple’s mutual concern and interest. The control dimension is designed to cover various interpersonal strategies and techniques, which can be used by couples to impact the partner’s behavior. Finally, the values and norms influence all aspects of the couple’s functioning and determine what is acceptable within the dyad. The items are rated on a 4-point scale, ranging from 0 (’strongly agree) to 3 (’strongly disagree’). The scores are computed separately for the seven subscales by adding up the points for each item. Scoring for some items is reversed due to negative wording. The theoretical range of the scores for each dimension is from 0 to 12. Higher scores indicate more maladaptive patterns of a given dimension of family functioning. The authors reported internal reliability coefficients (alphas) of 0.86-0.95, and test-retest reliabilities of 0.57-0.66 (26, 27).

Beck Depression Inventory (BDI) is a self-report measure designed to measure severity of depressive symptoms as experienced over an indicated period of time (29). The Polish adaptation of the original version of this instrument was used in the study, previously validated in Polish samples (30). BDI contains 21 multiple-choice items, each scored on a 4-point scale, from 0 to 3. The total score ranges from 0 to 63 with higher scores implying more severe depressive symptoms (29, 30). Several proposals of the BDI threshold for depression were suggested; in this study, we assumed the score of 12 as a cut-off for clinical depression (31). Most studies reported Cronbach’s alpha reliability coefficients higher than 0.75, the mean alpha coefficient of 0.88 was reported for psychiatric samples, and 0.82 for non-psychiatric samples (32).

Personal Data Sheet was developed for the purpose of this study. It was used in two versions - for infertile and fertile women and it served to collect selected socio-demographic data, including age, place of residence, educational level, and the relationship status and duration. The version for infertile women contained additional questions referring to clinical variables pertaining to infertility, such as the length of period the couples have been trying to conceive, treatment period, and a possible cause of infertility.

Statistical analyses were performed using the Statistical Package for the Social Sciences (SPSS; SPSS Inc., USA) for Windows 12.0. Descriptive statistics were calculated as frequencies/means and standard deviations for each variable. Based on the features of the data distribution, appropriate analyses were performed, including Pearson’s r correlation coefficients or Spearman’s rank correlation to analyze the relationships between the variables measured on continuous scales. Student’s t tests and Pearson’s chi-squared tests were used to test the between-the group differences for continuous and categorical variables, respectively. Multiple regression analysis was applied to identify statistically significant predictors of sexual satisfaction and sexual reactivity. P≤0.05 was accepted as the level of statistical significance.

## Results

### Participants’ characteristics

The socio-demographic characteristics and variables associated with infertility in the sample are shown in table 1. The cause of infertility was known in 41 women (82%) from the clinical group, and in nine cases, it remained unrecognized. The treatment duration was as follows: less than 6 months (48%), between 6 months to 12 months (10%), between 13 to 24 months (28%), and more than 24 months (14%).

**Table 1:** Socio-demographic characteristics and variables associated with infertility in the sample


	Infertile women		Control group	
	n	%	n	%

Age (Y)	≤20	1	2.0	3	6.0
	21-30	34	68.0	43	86.0
	31-40	15	30.0	4	8.0
Education	Secondary	13	26.0	29	58.0
	Higher	37	74.0	21	42.0
Origin	Urban	32	64.0	31	62.0
	Rural	18	36.0	19	38.0
Relationship duration (Y)	0.5-1	0	0	16	32.0
	2-4	25	50.0	23	46.0
	≥5	25	50.0	11	22.0
Time trying to conceive (Y)	1-2	21	42.0	-	-
	3-4	20	40.0	-	-
	≥5	9	18.0	-	-
Infertility reason	Known	41	82.0	-	-
	Unknown	9	18.0	-	-
Treatment duration (Y)	≤0.5	24	48.0	-	-
	0.5-1	5	10.0	-	-
	1-2	14	28.0	-	-
	>2	7	14.0	-	-


### Comparison of infertile women and the control group on psychological measures

Statistically significant differences were found between infertile women and fertile women with respect to sexual satisfaction and sexual reactivity. Significantly lower mean scores on all dimensions of sexual satisfaction (physical satisfaction, emotional satisfaction and satisfaction with control, P≤0.001) were found in infertile women as compared to the control group. The index of sexual reactivity (the total score on Mell-Krat Scale) was significantly lower (indicating more problems with sexual reactivity) in the sample of infertile women as compared to fertile women (P≤0.001). Ninety percent of infertile women and 26% of fertile women obtained the scores lower than the Mell-Krat cut-off score of 55, which might imply an elevated risk of dysfunctions in sexual reactivity. These results are presented in table 2.

With regard to the dyadic relationships, infertile women differed significantly from fertile women in the scores on six FAM-III subscales (task accomplishment, role performance, communication, affective expression, affective involvement, and values and norms). Infertile women were found to report higher scores on each of these subscales, compared to the control women. No statistically significant difference was observed with regard to the control subscale of FAM-III (P=0.30, Table 2).

A significant difference was observed between the infertile women and the control group with respect to severity of depressive symptoms. The mean BDI score was significantly higher in the group of infertile women indicating that they were more severely depressed (Table 2). Based on the BDI threshold score of 12, 39 (78%) of infertile respondents were within the range for depression [including 38 (76%) for moderate and 1 (2%) for severe depressive symptoms]. Only 1 (2%) women from the control sample obtained the scores within the range for depression, and the difference between the groups was statistically significant (χ²=60.26, df=3, P<0.001).

**Table 2:** Comparison of the mean scores on the SSS subscales, Mell-Krat Scale, FAM-III subscales and BDI in infertile women (n=50) and in the control group (n=50)


	Infertile women		Control group			
	M	SD	M	SD	t	P

Physical satisfaction (SSS)	34.36	6.18	42.58	6.71	-6.37	0.000
Emotional satisfaction (SSS)	9.66	3.94	13.54	4.22	-4.75	0.000
Satisfaction with control (SSS)	17.88	3.44	23.38	4.08	-7.29	0.000
Sexual reactivity (Mell-Krat Scale)	46.68	7.15	60.56	8.31	-8.95	0.000
Task accomplishment (FAM-III)	5.38	2.17	3.32	1.74	5.24	0.000
Role performance (FAM-III)	4.10	1.88	1.78	1.69	6.49	0.000
Communication (FAM-III)	3.72	1.81	2.58	2.03	2.97	0.004
Affective expression (FAM-III)	3.66	1.77	2.60	1.75	3.01	0.003
Affective involvement (FAM-III)	2.60	1.94	1.06	1.65	4.28	0.000
Control (FAM-III)	2.78	2.12	2.36	1.86	1.05	0.295
Values and norms (FAM-III)	3.54	2.03	1.64	1.72	5.04	0.000
Depressive symptoms (BDI)	16.64	6.45	2.44	2.44	14.56	0.000


The comparisons were made by means of Student’s t tests, with Cochran-Cox correction for heterogeneous variances. M; Mean, t; Statistic of Student’s t test, P; Significance level, SSS; Sexual satisfaction scale, FAM-III; Family assessment measure and BDI; Beck depression inventory.

### Psychological correlates of sexual satisfaction and sexual reactivity in infertile women and in the control group

In both samples, a pattern of negative correlations was found between physical sexual satisfaction and the qualities of the dyadic relationship, as measured by FAM-III (Table 3), indicating that higher physical sexual satisfaction was associated with better dyadic functioning (P≤0.05). However, in the sample of infertile women, the correlations of physical satisfaction with affective expression and control were non-significant, and overall the correlations were slightly lower than in the control group. Emotional sexual satisfaction was significantly associated with affective involvement (P≤0.001) and values and norms (P≤0.05) in infertile women, whereas with role performance and communication (P≤0.001) in the control group. Satisfaction with control over sexual life was unrelated to the dyadic functioning in the control group, and significantly associated with task accomplishment, role performance, affective involvement and values and norms (P≤0.001) in infertile women. Negative correlations were found between all three indexes of sexual satisfaction and depressive symptoms in the control group. Interestingly, these associations were not statistically significant in the sample of infertile women. There was no correlation between sexual reactivity and the aspects of the dyadic relationship or between sexual reactivity and depressive symptoms in either sample (Table 3).

**Table 3:** Pearson’s r correlation coefficients between sexual satisfaction and sexual reactivity with FAM-III subscales and the BDI total score in the samples of infertile (I) and fertile (F) women


	Physical satisfaction		Emotional satisfaction		Satisfaction with control		Sexual reactivityMell-Krat	
	I	F	I	F	I	F	I	F

Task accomplishment	-0.32*	-0.48**	-0.25	-0.23	-0.41**	0.09	-0.16	-0.09
Role performance	-0.32*	-0.47**	-0.20	-0.38**	-0.39**	-0.22	-0.10	-0.17
Communication	-0.31*	-0.54**	-0.08	-0.40**	-0.27	-0.18	-0.03	-0.14
Affective expression	-0.23	-0.51**	-0.06	-0.14	-0.16	-0.05	-0.09	-0.23
Affective involvement	-0.43**	-0.51**	-0.37**	-0.17	-0.43**	-0.18	-0.14	-0.10
Control	-0.15	-0.49**	-0.03	-0.12	-0.08	-0.04	-0.11	-0.18
Values and norms	-0.34**	-0.31**	-0.25*	-0.12	-0.38**	-0.01	-0.13	-0.09
Depressive symptoms (BDI)	-0.11	-0.35**	-0.12	-0.33**	-0.11	-0.32**	-0.09	-0.18


Higher scores on FAM-III (dyadic functioning) are indicative of more maladaptive functioning. *; P≤0.05, **; P≤0.001, FAM-III; Family assessment measure and BDI; Beck depression inventory.

### Socio-clinical correlates of sexual satisfaction and sexual reactivity in infertile women

Associations between sexual satisfaction and reactivity, and the relationship duration, period of trying to conceive and treatment period were assessed in the sample of infertile women (Table 4). Out of three indexes of sexual satisfaction, statistically significant negative correlations were found only for satisfaction with control. This aspect of sexual satisfaction correlated negatively with relationship duration and treatment period (P≤0.001). A statistically significant negative correlation was also observed between sexual reactivity and period of trying to conceive (P≤0.001).

### Prediction of sexual satisfaction and sexual reactivity- regression analysis

Multiple regression analysis was performed to identify the variables which are best predictors of sexual satisfaction and sexual reactivity in infertile women and in the control group. The following independent variables were entered into the regression model: socio-demographics (age, educational level, place of origin, and duration of the relationship) dyadic functioning and depressive symptoms. In the sample of infertile women, clinical variables (period trying to conceive and treatment period) were additionally entered into the regression model. Sexual satisfaction and sexual reactivity were used as dependent variables.

Regression analyses revealed different predictors of sexual satisfaction and sexual reactivity in each sample. Affective involvement and relationship duration were found to have a statistically significant predictive value for sexual satisfaction in infertile women, while communication, affective expression and depressive symptoms were significant predictors of sexual satisfaction in the control women.

Period of trying to conceive was the only significant predictor which explained approximately 15% of the variance in sexual reactivity in infertile women. None of the variables entered into the regression model were found to have a significant predictive value for sexual reactivity in the control group. The summary of the models obtained in stepwise multiple regression analysis is shown in table 5.

**Table 4:** Spearman’s rank correlation coefficients between sexual satisfaction and sexual reactivity, and relationship duration, period of trying to conceive and treatment period in the sample of infertile women


	Physical satisfaction	Emotional satisfaction	Satisfaction with control	Sexual reactivityMell-Krat

Relationship duration	-0.22	-0.13	-0.40**	0.03
Period of trying to conceive	-0.25	-0.05	-0.22	-0.32**
Treatment period	-0.19	0.02	-0.31**	-0.09


**; P≤0.001.

**Table 5:** Results of stepwise multiple regression analyses performed for sexual satisfaction and sexual reactivity (dependent variables) in infertile women and in the control group


	Infertile women					Control group			
Dependent variable	Predictor	Beta	R^2^	P	Dependent variable	Predictor	Beta	R^2^	P

Physical satisfaction	Affective involvement	-0.43	0.19	0.002	Physical satisfaction	Communication	-0.36		0.014
Emotional satisfaction	Affective involvement	-0.37	0.13	0.009	Emotional satisfaction	Affective expression	-0.31	0.35	0.033
Satisfaction with control	Affective involvement	-0.38	0.32	0.003	Satisfaction with control	Communication	-0.40	0.16	0.004
	Relationship duration	-0.38		0.003		Depressive symptoms	-0.32	0.10	0.024
Sexual reactivity	Period of trying to conceive	-.039	0.15	0.005					


For each dependent variable, only these predictors are shown in the table which reached the statistical significance. R^2^; Coefficient of determination and P; Significance level.

## Discussion

Previous studies have indicated that infertility might have an extensive, deleterious impact on sexual satisfaction (3, 13, 14) and sexual functioning (33, 34). In our sample of infertile women, we found the evidence for an elevated risk of sexual dysfunctions: as many as 90% of infertile women, as compared to 26% of women from the control group, reported the scores on the Mell-Krat Scale indicative of dysfunctions in sexual functioning. The numbers of infertile women at risk for sexual dysfunctions we found in our study are even higher than those reported in other studies, e.g. 40% in Millheiser et al.’s (35) study or 61,7% in Oskay et al.’s (5) study. This difference may be due to different criteria for detecting sexual dysfunctions applied in the studies, as the latter studies used for this purpose Female Sexual Function Index, whereas we applied the Mell-Krat Scale which is more commonly used in Poland. The cut-off score of 55 on the Mell-Krat Scale is recommended in Polish sexological literature for detecting increased risk of sexual dysfunction. However, the rate of sexual dysfunctions in the control group from our study was consistent with the data for the general population of Polish women, in which 25% of Polish women complained of lowered sexual needs (36). Even if the prevalence of sexual dysfunctions we found in the sample of infertile women is slightly overestimated, it is nevertheless much higher than in the control group, which altogether adds to the previously reported evidence for the link between infertility and sexual dysfunctions in women.

In the present study, infertile women reported significantly lower levels of sexual satisfaction as well as more maladaptive patterns of couple functioning, compared to the control group. Infertile women scored significantly lower on all three dimensions of sexual satisfaction and significantly higher (maladaptive) on all six domains of dyadic functioning (task accomplishment, role performance, communication, affective expression, affective involvement, and values and norms). Based on these findings, one can speculate that infertility may be considered as a specific crisis, in which the quality of sexual functioning is closely associated with treatment procedures, for example timing of the intercourse around the ovulatory cycle. This ‘crisis’ may also be a stressful experience because of the long-lasting treatment and its inconveniences, especially when the long-term treatment is unsuccessful. Infertility crisis may induce stress in affected couples, which in turn may have a deleterious impact on both sexual activity and dyadic functioning. It was previously found that experienced stress was negatively correlated with sexual behavior and satisfaction, and that higher self-reported stress in daily life was associated with lower level of relationship satisfaction (37).

The present study found significantly higher rates of depressive symptoms in infertile women, as compared to the control group. More than threequarter of our infertile women had the scores on BDI within the range of the risk for clinical depression. The mean levels of depressive symptoms were also significantly higher in infertile women than in the control group. Other studies reported similar results, demonstrating that infertile women have elevated mean levels of depressive symptoms (38), more than three times higher odds ratios for dysthymia compared to the women without infertility (4) and increased rates of depression (18). It should be remembered, however, that our sample of infertile women contained slightly more participants aged above 30 (30%) than the control group (8%), which might also affect the higher rates of depression in the sample of infertile women.

Previous studies suggested the bidirectional relationship between depressive symptoms and sexual/ marital satisfaction, so that high levels of depressive symptoms might be considered as a predictor of low sexual satisfaction (39), as well as marital dissatisfaction can be regarded as a risk factor for depressive symptoms (40). However, in the present study, a significant relationship between sexual satisfaction and depressive symptoms was found in the control group but not in the women with infertility. This suggests that while in healthy women sexual satisfaction is negatively associated with levels of depressive symptom, in infertile women depression levels (even though significantly elevated) do not relate to levels of sexual satisfaction. It is of interest that one study found infertile couples to exhibit more resilience (resistance to psychosocial stress) than fertile couples, and the levels of resilience are correlated positively with quality of life (41). If resilience works as a protective factor in infertile individuals, this can shed more light on our findings, suggesting resilience may buffer the relationship between depressive symptoms and sexual satisfaction in infertile women from our sample.

It was also reported that sexual satisfaction may depend on relationship duration, with women exhibiting greater sexual satisfaction later rather than earlier in the relationship (42). On the other hand, ambiguous data were reported for the association between sexual satisfaction and treatment duration (43, 44). The results obtained in this study showed that both relationship duration and fertility treatment duration were negatively correlated with sexual satisfaction, particularly with satisfaction with control over sexual activity.

Likewise, a pattern of negative correlations was found between physical sexual satisfaction and the qualities of the dyadic relationship in both samples, indicating that sexual satisfaction is closely related to dyadic adjustment, irrespective of the fertility status. It is of note, however, that the correlations between physical sexual satisfaction and dyadic functioning were generally stronger in the control group than in the women with infertility. This suggests that infertility may be a mediator of the relationship between physical sexual satisfaction and dyadic functioning. This also means that sexual satisfaction and dyadic functioning may be less dependent on each other in infertile couples and instead be affected to a greater extent by other factors such as those related to infertility.

No correlation was observed between sexual reactivity and dimensions of dyadic functioning in infertile and control group. Negative correlations were revealed between sexual reactivity and the period of trying to conceive. It seems essential that a longer history of efforts to conceive is associated with a longer history of intercourses primarily aimed at fertilization and a longer history of frustration (failed attempts to get pregnant), which may result in decreased levels of libido and weaker pre-coitus arousal in infertile women (45) which may in turn lead to dysfunctions in their sexual life. These findings were partially supported by results obtained in stepwise multiple regression analysis, which was performed to better understand the relationship between sexual satisfaction and sexual reactivity, and dyadic functioning. Multiple regression analyses revealed different predictors of sexual satisfaction and sexual reactivity in each sample. Indeed, the period of trying to conceive was found to have a predictive value for sexual reactivity in infertile women, implying that a longer history of unsuccessful attempts to conceive may induce discouragement and impatience that lead to a decline in sexual functioning. Different predictors of sexual satisfaction in each group (affective involvement and relationship duration in infertile women and communication, affective expression and depressive symptoms in the control group) might reflect the differences of the life situation in both groups. Perhaps, in the face of infertility crisis, affective involvement is more important than other aspects of dyadic functioning for sexual functioning, since it helps to bind the couples emotionally, and feelings of closeness, togetherness, and fulfillment of safety and understanding needs may be especially important in the case of infertility.

In summary, the relationships between sexual satisfaction and reactivity, and dyadic functioning were evaluated in the present study. The negative pattern of correlations was found between sexual satisfaction and dyadic functioning in either sample, indicating that sexual satisfaction is closely related to partner adjustment. It seems that sexual satisfaction is an important aspect of partner functioning and it might form a bidirectional link with dyadic functioning. On the one hand, impairment of sexual satisfaction may decrease dyadic adjustment. On the other hand, lower levels of partner functioning might lead to lower sexual satisfaction. Furthermore, the treatment period and relationship duration were found to be related to sexual satisfaction, so that infertile women whose relationships lasted longer and those whose treatment had been unsuccessful for a longer period were less satisfied with their sexual relationships.

In this study, infertile women were less satisfied with their sexual relationships and they revealed more dysfunctions of sexual functioning, compared to the control group. They also reported more depressive symptoms than the control sample. These findings are supported by previous studies in which infertile women were found to reveal higher levels of depression and anxiety (18) and had more sexual dysfunctions than fertile women (5). Interestingly, different variables were found to have a predictive value for sexual satisfaction in both samples (affective involvement and relationship duration for infertile women and communication, affective expression and depressive symptoms for the control group), which might be attributed to the differences of the life situation in each group.

Some limitations of this study must be mentioned. First, the sample was not cross-cultural or recruited from multiple centers; therefore, the results we obtained may be specific only for the Polish cultural background from which the sample originated. Second, the control group was recruited based on the participants’ self-reports of no problems with fertility. However, since the women did not intend to get pregnant, some of them might be unaware of their fertility status. Anyway, this limitation is very difficult to avoid, if childless women are matched for the infertile women. Third, the size of the sample was relatively small, which might affect the power of some statistical tests employed. For instance, the number of the independent variables entered into regression models might be excessive for the actual sizes of the subsamples. Finally, the instruments we used also carry cultural specificity, for instance, Mell- Krat Scale is commonly used in Poland; however, is relatively unknown abroad, which makes it difficult to make comparisons with other studies. All these limitations must be taken into consideration when generalizing and interpreting the results of this study.

## Conclusion

The results of this study provide evidence for a detrimental impact infertility can exert on sexual satisfaction, sexual reactivity and dyadic functioning. Moreover, it is linked to elevated depressive symptoms in infertile women sample, especially when fertilization treatment is unsuccessful for a prolonged time.
